# Bioresorbable Scaffolds: Current Evidence and Ongoing Clinical Trials

**DOI:** 10.1007/s11886-012-0295-5

**Published:** 2012-07-19

**Authors:** Christos V. Bourantas, Yaojun Zhang, Vasim Farooq, Hector M. Garcia-Garcia, Yoshinobu Onuma, Patrick W. Serruys

**Affiliations:** Thoraxcenter, Erasmus Medical Center, ‘s-Gravendijkwal 230, 3015 CE Rotterdam, The Netherlands

**Keywords:** Coronary artery disease, Stents, Bioresorbable scaffolds, Vascular restoration therapy

## Abstract

Bioresorbable scaffolds (BRS) represent a novel approach in coronary stent technology. In contrast to the metallic stents, they provide transient scaffolding, thereby safeguarding early vessel patency and acute gain. Subsequently a process of “decomposition” occurs, that results in the complete absorption of the scaffold. This reduces the risk of late complications, allowing the vessel to maintain its integrity and physiological function. This unique ability has attracted interest and nowadays several BRS are available. The aim of this review article is to describe the advances in the field, present the evidence from the preclinical and clinical evaluation of these devices, and provide an overview of the ongoing clinical trials that were designed to examine the effectiveness of BRS in the clinical setting.

## Introduction

Bioresorbable scaffolds (BRS) have been heralded as the fourth revolution in interventional cardiology as they introduce a novel concept in the treatment of coronary artery disease. These devices have the unique ability to permit the restoration of vascular physiology and integrity as they provide a temporary scaffold that is necessary to maintain the patency of the vessel after intervention, and then they gradually dissolve, liberating the vessel from its cage [1•, 2, 3]. Thus, it is expected that BRS will potentially overcome the limitations of the traditional stents, such as the risk of late stent thrombosis, neoatherosclerosis, and the local inflammation caused by the presence of a foreign body [4, 5]. In addition, BRS will allow a potential surgical revascularization of the treated segment whereas traditional stents often preclude this option.

**Fig. 1:**
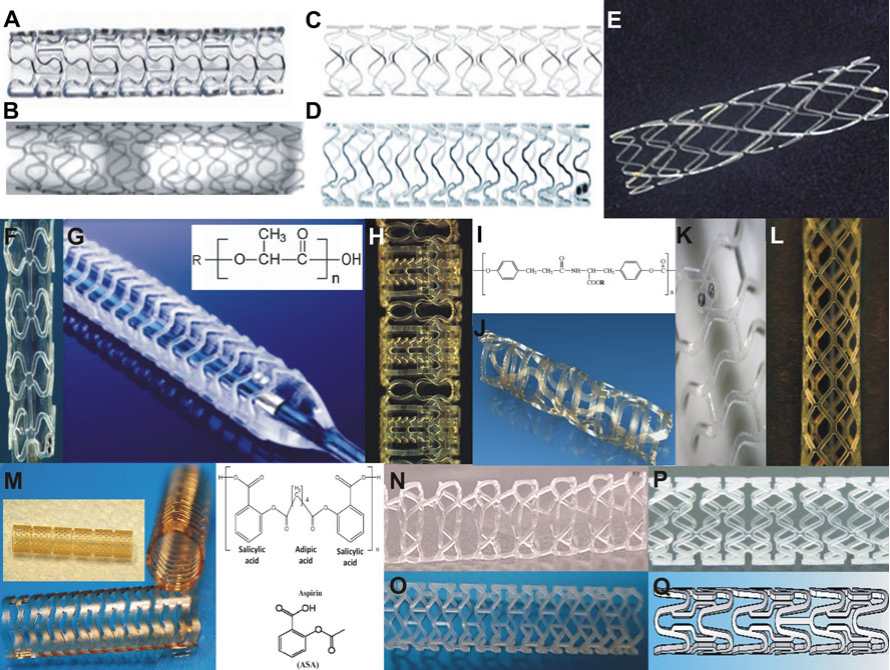
Developed BRSs. (**a**) Absorbable metallic stent (AMS) 1, (**b**) AMS 2, (**c**) drug-eluting AMS (DREAMS) 1, (**d**) DREAMS 2, (**e**) Igaki-Tamai stent, (**f**) Abbott Vascular BRS 1.0, (**g**) Abbott Vascular BRS 1.1 and chemical structure of the PLLA, (**h**) Reva Medical BRS revision 1, (**i**) molecular composition of the Reva Medical BRS and (**j**) second revision of the REVA Medical BRS, (**k**) DeSolve BRS, (**l**) ART BRS, (**m**) first and second generation IDEAL scaffold and chemical structure of the polymer used, (**n**) Acute BRS, (**o**) Amaranth PLLA, (**p**) Xinsorb, and (**q**) Sahajanand BRS. The images for the Igaki-Tamai stent and for the IDEAL biostent were reprinted by permission of Lippincott Williams & Wilkins from: Circulation. 2011;123:779-97 [34], while the image for the Sahajanand BRS was reprinted by permission of Edizioni Minerva Medica from: Minerva Cardioangiol. 2009;57:537–65 [18]

The potential unique advantages of this rapidly developing technology have lead to a drive towards the development of several types of BRS by industry. Hence, today numerous scaffolds are available with different compositions (eg, metallic alloy or polymer), strengths, and weaknesses (Fig. 1). Some of these are under development, some undergoing pre-clinical evaluation, while others have already been implanted in humans. The aim of this review article is to describe the advances in this field, present the evidence stemming from the evaluation of available BRS, and provide a synopsis of the ongoing clinical trials designed to examine the effectiveness of these devices in the clinical arena (Table 1).

**Table 1:** Aim, design, and primary and secondary end-points of the ongoing clinical studies

Device	Name	Aim	Design	Number of patients	Follow-up	Primary end-points	Secondary-end points
DREAMS	BIOSOLVE-I	Assess the safety and efficacy of DREAMS	Prospective, open label	46	3 years	1. Target lesion failure at 6 and 12 months	1. LLL, binary restenosis and scaffold stenosis at 6 and 12 months
							4. Target lesion failure at 6, 12, 24, and 36 months
Abbott Vascular BRS 1.1	ABSORB Cohort B	Assess the safety and effectiveness of the BRS 1.1	Prospective, open label	101	5 years	-	1. LLL at 6 months 1, 2, and 3 years
							2. Diameter stenosis at 6 months, 1 and 2 years
							3. MACE and TLR at 30 days 6, 9 months 1, 2, 3, 4, and 5 years
Abbott Vascular BRS 1.1	ABSORB Extend	Assess the safety and performance of the BRS 1.1	Registry	1000	5 years	1. Acute success	1. Scaffold and lumen area, MLA and struts apposition assessed by OCT at 2 years
						2. No other primary end points – all outcomes are of equal weight	2. LLL, MLD, % diameter stenosis on angiography at 2 years
							4. IVUS evaluation including vessel and scaffold area, MLA, volume obstruction
							3. MACE and TLR at 30, days, 6 months 1, 2, and 3 years
Abbott Vascular BRS 1.1	ABSORB II	Compare the safety and efficacy of the BRS 1.1 to Xience Prime	Prospective, randomized control trial	501	3 years	1. TRL at 6 months	1. Device – procedural success
						2. Vasomotion of the treated lesion at 2 years	2. Death, MI, MACE at 30, 180 days, 1, 2, and 3 years
							3. TLR, TVR, scaffold thrombosis at 30, 180 days, 1, 2, and 3 years
Abbott Vascular BRS 1.1	ABSORB Physiology	Evaluate the effect of the BRS 1.1 and the Xience DES on vessel physiology postprocedure and at 2 years follow-up	Randomized single blind	36	2 years	1. Coronary artery endothelial response postprocedure	1. Vessel impedance, compliance, distensibility and wall shear stress, postprocedure and at 2 years follow-up
							2. Cardiac and all cause mortality, MI, MACE, TVR, TLR and stent thrombosis at 6 months, 1 and 2 years
ReZolve	RESTORE	Examine the safety and efficacy of the ReZolve BRS	Prospective, open label	50	5 years	1. TLR at 6 months	1. Procedural success
						2. QCA and IVUS measurements at 12 months	2. LLL, restenosis rate, MLD, neointima volume at 12 months
							3. MACE at 12 months
ReZolve	ReZolve CE Mark	Demonstrate non-inferiority of the ReZolve BRS against a DES	Randomized, trial	350	5 years	1. Clinical follow-up at 1, 6, and 12 months and then annually for up to 5 years	1. LLL
						2. Invasive imaging at 9 and 12 months	2. MACE
DeSolve	DeSolve I	Evaluate the safety and efficacy of DeSolve BRS	Prospective, open label	16	5 years	1. LLL at 6 months	1. Device and procedural success
							2. MACE, TLR, TVR and stent thrombosis at 1, 6, 12 months, 2 and 5 years
							3. OCT measurements at 6 months
DeSolve	DeSolve NX	Evaluate the safety and efficacy of DeSolve BRS	Prospective, open label	120	5 years	1. Procedural success	-
						2. MACE at 1, 6, and 12 months, 2, 3, 4, and 5 years	
						3. LLL at 6 months	

LLL, late lumen loss; BRS, bioresorbable scaffold; MACE, major adverse cardiac events; TLR, target lesion revascularization; MLA, minimal lumen area; MLD, minimal luminal diameter; OCT, optical coherence tomography; IVUS, intravascular ultrasound; MI, myocardial infarction; TVR, target vessel revascularization; QCA, quantitative coronary angiography; CE, Conformité Européenne; DES, drug eluting stent

## BRS Assessed in Clinical Setting

### Igaki-Tamai Stent; the 1st BRS

The Igaki-Tamai stent (Kyoto Medical Planning Co, Ltd, Kyoto, Japan) is the first BRS implanted in humans. It is made of poly-L-lactic acid (PLLA) monofilament and does not contain any antiproliferative drug. The scaffold has a zig-zag helical coil design with 2 radiopaque markers at the proximal and distal end of the stent. Initially, stent implantation required an 8F guide catheter and was performed using heated angio contrast media at 80 °C. *In vitro* experiments have shown that the device expands by itself to its original size within 0.2 seconds when it is heated to 70 °C, while at a human’s temperature stent expansion takes 20 minutes.

The Igaki-Tamai stent was implanted for the first time in 1998, and in 2000 a report was published that demonstrated the feasibility and effectiveness of the device [6]. Fifteen patients (19 lesions) were successfully treated with 25 scaffolds. Coronary angiography and intravascular ultrasound (IVUS) performed the day after device implantation revealed the absence of significant recoil. There was only 1 target lesion revascularization (TLR) at 6 months follow-up. Quantitative coronary angiography (QCA) performed at 3 and 6 months demonstrated a mean diameter stenosis of 33 ± 14 % and 33 ± 18 %, respectively. IVUS examination showed that at 3 months the stent area increased and the lumen area decreased (from 7.42 ± 1.51 mm^2^ to 8.18 ± 2.42 mm^2^ and from 7.42 ± 1.51 mm^2^ to 5.67 ± 2.42 mm^2^, respectively) but they did not further change at 6 months follow-up (stent area: 8.13 ± 2.52 mm^2^; lumen area: 5.63 ± 2.70 mm^2^).

Recently, Nishio et al reported the long term outcomes (>10 years) after the implantation of the Igaki-Tamai stent [7••]. In this study 50 patients (63 lesions) were treated with 84 BRS. One sub-acute scaffold thrombosis occurred during hospitalization and was attributed to discontinuation of the antiplatelet treatment due to an acute hemorrhagic gastric ulcer. At 10 years follow-up 7 deaths (1 of unknown cause and 6 due to noncardiac causes) and 3 additional myocardial infarctions (MI) were reported (1 lesion related and 2 nonlesion related). The TLR rate was 28 % (14 cases). The late lumen loss (LLL) was 0.91 ± 0.69 mm at 6 months, but improved to 0.67 ± 0.45 mm at 1 year and was only 0.59 ± 0.50 mm at 3 years follow-up. Grayscale serial IVUS examination performed in 18 patients showed that the minimum lumen area decreased at 6 months (from 6.19 ± 2.26 mm^2^ postprocedure to 4.23 ± 1.82 mm^2^), and then increased (4.95 ± 1.79 mm^2^) at 3 years follow-up. Conversely, the scaffold area increased at 6 months and at 1 year follow-up (from 7.63 ± 2.69 mm^2^ postprocedure to 8.13 ± 2.63 mm^2^ at 6 months and 7.95 ± 2.65 mm^2^ at 1 year), with the scaffold no longer detectable after 3 years. Echogenicity analyses of the serial IVUS data was performed in 13 lesions, and showed a significant increase in the echogenicity of the scaffolded vessel after device implantation, with a progressive reduction in echogenicity at 1 year, and returning to the pre-implantation values at 3 years follow-up, thereby indicating complete resorption of the scaffold.

Although the abovementioned results were promising, the device failed to progress as it required a larger guide catheter for implantation and heated contrast, the latter being a potential cause of concern in causing local vessel wall injury [8]. Kyoto Medical has recently improved the design of the device, which can now be implanted through a 6F guide catheter without the need for a heated contrast agent. The second generation Igaki-Tamai stent is currently undergoing preclinical evaluation in Germany.

### Magnesium Metallic Stent

The absorbable metallic stent (AMS) (Biotronik, Berlin, Germany) is composed of magnesium and rare earth metals, and is the only bioresorbable metallic stent implanted in humans. The device has a high mechanical strength and similar properties to the other metallic stents. Experimental studies have demonstrated that it has antithrombotic properties attributed to the electronegative charge produced during the degradation of the device [9]. The majority of the resorption process is completed within 2 months and produces inorganic salts without causing significant inflammatory response [10].

The feasibility and efficacy of the first generation AMS (AMS 1) was examined in the PROGRESS AMS trial. Sixty three patients with a single de novo lesion were included and received 71 scaffolds. There was a high incidence of TLR (45 %) at 12 months and an increased LLL on angiogram performed at 4 months follow-up (1.08 ± 0.49 mm). At this time point, the vasomotor function was assessed in 5 treated segments and appeared restored [11]. IVUS at 4 months follow-up showed almost complete resorption of the device and a significant reduction in luminal dimensions. Forty five percent of this reduction was attributed to neointima formation, 42 % to negative remodeling, and 13 % to an increase in the plaque area outside the stent. The negative remodeling was attributed to an early reduction of the scaffold’s radial force that was due to the fast resorption of the device. Thus, the AMS 1 was modified using a different magnesium alloy, which provided increased radial strength and prolonged the duration of the resorption process. Preclinical evaluation in animal models confirmed the effectiveness of the second generation AMS [12]. Before being implanted in humans, the device underwent further modifications with the incorporation of paclitaxel elution and a biodegradable matrix that could control the release of the antiproliferative drug (AMS 3). The drug-eluting AMS (DREAMS) was tested in clinical setting in the BIOSOLVE-I study. In this prospective, multi-center, first-in-man trial, 46 patients with a single de novo lesion with a reference diameter between 3.0–3.5 mm were included. Forty seven DREAMS were successfully implanted. The TLR rate at 6 months was 4.3 %, and no other cardiac events were reported. The LLL was 0.64 ± 0.50 mm at this time point. A restoration of vessel geometry was also noted at 6 months, with the angulation of the treated segments reported to increase from 14.9 ± 12.0° immediately postprocedure to 26.1 ± 15.9° at late follow-up (lesion angulation at baseline: 31.4 ± 21.2°) [13].

DREAMS was further modified to create the next generation DREAMS. DREAMS 2 has radiopaque markers at both ends (made from tantalum) and sirolimus elution instead of paclitaxel. Preclinical evaluation of the device in porcine models demonstrated a better endothelization and reduced inflammation in the first 2 months post-implantation comparing to DREAMS 1. The device has not yet been implanted in humans [14].

### Abbott Vascular BRS

The Abbott Vascular BRS (Abbott Vascular, Santa Clara, CA, USA) is made from semicrystalline PLLA coated with the amorphous poly-D, L-lactide (PDLA) polymer, which contains and controls the release of the antiproliferative drug everolimus. Device degradation requires 2–3 years and includes hydrolysis of the PLLA and PDLA. The outcome of this process is the formation of small molecules of lactic acid which are phagocytosed by macrophages when their diameter becomes <2 μm. The resorption is completed with the catabolism of these molecules in the Krebs cycle.

The first Abbott Vascular BRS device (revision 1.0) was investigated in humans in the ABSORB Cohort A study. In this single-arm, prospective, open-label study, 30 patients with single de novo coronary artery disease and stable or unstable angina were enrolled [15]. During the follow-up period, there was only 1 non-Q wave MI, while the reported LLL was 0.43 ± 0.37 mm at 6 months and 0.48 ± 0.28 mm at 2 years. The vasomotor function was restored at 2 years [16]. IVUS examination at 6 months revealed scaffold shrinkage (from 6.94 ± 1.70 mm^2^ to 6.29 ± 1.47 mm^2^) which appeared to be affected by the composition of the plaque and was more intense in fibrofatty and lipid rich lesions [17]. To overcome this drawback, the BRS was re-designed. The improved revision, 1.1, had a different design with its struts having an in-phase hoop, with straight links arrangements to provide to the scaffold an increased radial support. In addition, the polymer in the updated version was processed in such a way so as to give the scaffold additional mechanical strength [18].

The performance of the BRS 1.1 was tested in the ABSORB Cohort B study. This multicenter, single-arm trial recruited 101 patients who had single or 2 vessel de novo coronary artery disease. All patients were treated with a 3 × 18 mm BRS. The studied population was divided into 2 groups. The first had invasive follow-up assessment (QCA, IVUS, IVUS palpography, IVUS virtual histology, IVUS echogenicity, and optical coherence tomography [OCT], which was optional) at 6 months and at 2 years, and the second group had similar assessments at 1 and at 3 years. Computed tomographic coronary angiography (CTCA) was performed in both groups at 18 months follow-up.

In group 1 there was only 1 TLR at 6 months, while the LLL was 0.19 ± 0.18 mm [19]. At 2 years the LLL was 0.27 ± 0.20 mm [20]. Follow-up IVUS examination demonstrated a reduction in the mean lumen area at 6 months (6.60 ± 1.22 mm^2^ vs 6.37 ± 1.12 mm^2^, *P* < 0.005). Similar results were reported in serial OCT examinations performed in 23 patients: the lumen was decreased at 6 months (7.23 ± 1.24 mm^2^ vs 6.07 ± 1.39 mm^2^, *P* < 0.001) with no further changes at 2 years (5.99 ± 1.61 mm^2^, *P* = 0.26). On the other hand, the scaffold area was reported to progressively increase during follow-up (7.47 ± 1.18 mm^2^ at baseline, 7.70 ± 1.34 mm^2^ at 6 months, and 8.34 ± 1.83 mm^2^ at 2 years) [19].

At the time of writing the invasive follow-up assessment of group 2 has not yet been completed. The 1 year follow-up results, which were recently published, are similar to those reported in group 1. Two non-Q wave MI and 2 TLR occurred within the first year, while the QCA showed a LLL of 0.27 ± 0.32 mm. OCT examination at 1 year demonstrated a significant reduction in the lumen area and an increase in the scaffold area [21].

Apart from the ABSORB Cohort B trial that it is underway, 3 more studies have recently commenced: the ABSORB Extend, the ABSORB Physiology, and the ABSORB II. The ABSORB Extend is a worldwide registry that aims to recruit 1000 patients with de novo single or 2 vessel disease and report the safety and efficacy of the device. In this registry a 2.5 mm BRS was introduced thus, allowing for the examination of the feasibility of a BRS in small vessels (reference diameter between 2.0 mm and 3.3 mm). In addition, patients with long lesions will be also included, and hence we will be able to evaluate the potential safety of overlapping devices. The ABSORB Physiology study aims to assess the acute and long-term effect of a BRS 1.1 compared with a conventional metallic drug-eluting stent, in terms of impact on coronary physiology. In this randomized trial 36 patients will be included and will be followed up for 2 years. The following variables will be evaluated: vascular compliance, distensibility, endothelial responsiveness (defined as change in vessel diameter during pacing, hand grip, and acetylcholine injection), and changes in the shear stress distribution, after device/stent implantation, and at 2 years follow-up. The ABSORB II is a prospective, randomized control trial that aims to compare the safety and efficacy of the BRS 1.1 vs the Xience prime stent. Five hundred one patients with stable angina and single or 2 vessel disease will be recruited and randomized on a 2:1 basis to BRS 1.1 and Xience prime stent implantation. The study has recently started and is expected to be completed by 2015.

### Reva Medical BRS

The Reva Medical BRS (Reva Medical Inc, San Diego, CA, USA) is a tyrosine poly (desamino tyrosyl-tyrosine ethyl ester) carbonate radiopaque stent. The first revision had a unique slide and locking design that provided flexibility and strength, eliminated the device hinge points and the polymer’s strain, and maintained the acute lumen gain after device deployment. The resorption process includes hydrolysis of the polymer and is completed between 18–24 months.

The performance of the device was examined extensively in animal models with initial results proving promising [22, 23]. Hence, the company proceeded to conduct the first-in-man trial, the RESORB study, which included 27 patients. The immediate post-procedure results showed an increase in the minimal lumen diameter from 0.88 ± 0.39 mm to 2.76 ± 0.39 mm, suggesting excellent scaffold expansion. Follow-up intravascular imaging revealed the absence of vessel shrinkage (external elastic lamina: 15.5 ± 4.0 mm^2^ at baseline and 15.3 ± 3.1 mm^2^ at follow-up). At 12 months there was a high event rate with 18 reported TLR, 3 of which lead to a non-Q wave MI [24]. These poor outcomes were attributed to focal mechanical failures of the device.

Thus, the scaffold was re-designed resulting in the second generation BRS. The updated ReZolve device had a stronger resilient polymer and a novel slide and spiral lock mechanism that provides better radial support and reduces vessel recoil. The new scaffold has a better crossing profile, can be implanted with the use of a 6F guide catheter (the previous generation device required a 7F guide), and instead of paclitaxel it has sirolimus elution. After successful preclinical evaluation, the Rezolve BRS is undergoing clinical evaluation in the RESTORE trial. This study aims to recruit 50 patients with de novo coronary artery disease and examine the safety and effectiveness of the new scaffold. The recruitment started in December 2011, and the studied population will be followed for 5 years [25]. The primary and secondary end-points are illustrated in Table 1. Apart from the RESTORE trial, there is a plan for the conduction of a further trial, the ReSolve CE Mark study, which is expected to commence later this year. This trial aims to provide the device with *Conformité Européenne* (CE) mark approval, and will randomize 350 patients on a 2:1 basis to treatment with the ReZolve BRS against a commercially available metallic drug-eluting stent.

### IDEAL BRS

The IDEAL (Xenogenics Corp, Canton, MA, USA) has 2 components: the backbone and the drug layer. The backbone of the device is made from polylactide anhydride mixed with a polymer of salicylic acid and sebacic acid linker, and is designed to provide radial strength. The drug layer consists of salicylate that controls the release of the antiproliferative drug sirolimus. The presence of salicylic acid provides the device with anti-inflammatory properties, which have been confirmed in preclinical studies [26]. The safety and the efficacy of the scaffold were tested in humans in the Whisper study. In this prospective, open label trial, 11 patients underwent BRS implantation and were followed-up for 18 months. The results were reported in EuroPCR in 2009 [27]. Although there was no scaffold shrinkage, there was a reduction in lumen area due to insufficient neointima suppression, which was attributed to inadequate drug dosing and to the rapid release of the sirolimus elution.

The second revision of the IDEAL BRS has a higher drug dose, a slower release rate, and in contrast to the first generation device that required an 8F guide catheter, it can be implanted through a 6F guide catheter. The device is currently undergoing preclinical evaluation [28].

### DeSolve BRS

DeSolve BRS (Elixir Medical, Sunnyvale, CA, USA) features a fully bioresorbable polymer (PLLA-based) scaffold that is coated with a PLLA-based resorbable polymer-drug matrix. It contains 2 antiproliferative drugs: novolimus at a dose of 5 mcg/mm and myolimus at a dose of 3 mcg/mm. The device was evaluated for the first time in humans in the DeSolve 1 study. Fifteen patients were enrolled in this feasibility trial and underwent percutaneous coronary intervention with the DeSolve BRS. Patients’ recruitment has been completed and the 6 months follow-up has begun and will be reported later this year. Preliminary follow-up imaging results have shown low neointima hyperplasia and no evidence of scaffold shrinkage [29]. In addition, the DeSolve NX study has recently commenced and aims to evaluate the safety and effectiveness of the BRS in 150 patients. The study design and the primary and secondary end-points are shown in Table 1. There is also a plan in the pipeline for the conduction of the DeSolve NX II pivotal trial that will examine the safety and efficacy of the device in a broad number of patients, so as to potentially acquire CE mark approval.

## BRS Undergoing Pre-clinical Evaluation

### ART BRS

The ART BRS (Arterial Remodeling Technologies, Noisy le Roi, France) is manufactured from a PLLA amorphous polymer. The device does not have drug elution, is 6F compatible, and provides vessel transient scaffolding for 5–7 months. Full resorption occurs within 18 months.

The performance of the BRS was compared with the Multilink bare-metal stent (Abbott Vascular, Santa Clara, CA, USA) in rabbit and porcine models. More than 250 devices were implanted. No MACEs were reported. The LLL was higher in the ART group in the first weeks, thereafter it began to decrease due to a “positive BRS remodeling,” and at 3 months the LLL in the ART scaffold was comparable to that observed in the Multilink bare-metal stent [30]. In view of these promising results, the company is currently designing the first-in-man study, “ART-FIM CE Mark,” which aims to assess the safety and feasibility of the device in humans and is expected to start this year.

### Amaranth

The Amaranth BRS (Amaranth Medical Inc, Mountain View, CA, USA) is composed of a PLLA polymer and does not contain an antiproliferative drug. The resorption process takes approximately 1–2 years.

The device is currently undergoing preclinical evaluation. The first results demonstrated that the BRS has excellent deliverability and radial strength post deployment. The LLL at 1, 3, and 6 months follow-up was similar in animals receiving the Amaranth BRS and in animals implanted with a Liberte bare-metal stent (Boston Scientific, Natwick, MA, USA). At all time points, the neointima proliferation, assessed by OCT, was smaller in the Amaranth BRS compared with the Liberte. The company is planning to commence this year the first-in-man study, which will include 30 patients and will compare the device with a commercially available bare-metal stent.

### Xinsorb BRS

The Xinsorb BRS (Huaan Biotechnology Group, Laiwu, China) is made by PLLA and is covered by sirolimus elution. The efficacy of the device has been recently examined in preclinical setting, and compared with the Excel drug-eluting stent (JW Medical System, Weihai, China), which has the same antiproliferative drug. In a small study recently reported in Transcatheter Cardiovascular Therapeutics 2011, 16 BRS, and 16 metallic stents were implanted in porcine arteries. No difference in the percent area stenosis (18.6 ± 5.2 % vs 21.4 ± 7.2 % and 24.5 ± 4.7 % vs 27.7 ± 5.6 %, *P* > 0.05) or in the inflammation score (0.84 ± 0.15 vs 0.74 ± 0.10 and 0.93 ± 0.26 vs 0.88 ± 0.10; *P* > 0.05) were reported between the 2 devices at 30 and 90 days follow-up [31]. These preliminary results are promising; however, further preclinical examination is necessary before conducting a first-in-man trial.

### Acute BRS

The Acute BRS (Orbus Neich, Fort Lauderdale, FL, USA) is different from the other BRS as it incorporates + CD34 antibodies for endothelial progenitor cells capture, which are expected to expedite the endothelial coverage of the BRS [32]. The device also has sirolimus elution, while its backbone is composed of 3 biodegradable polymers (poly-L lactic, poly-D lactic, and poly-L-lactide-co-ε-caprolactone) which provide the required radial strength. The device is currently undergoing preclinical evaluation in porcine models. The first angiographic and intravascular imaging results showed an optimal device implantation without evidence of fracture [33]. An interim update is expected at EuroPCR 2012.

## Other BRSs

Apart from the abovementioned BRS there are several other devices that are currently under development. These include the Sahajanand BRS (Sahajanand Medical Technologies Pvt Ltd, Surat, Gujarat, India), the Avatar BRS (S3V; Vascular Technologies Pvt Ltd, Bangalore, Karnataka, India India), the MeRes BRS (Meril Life Science, Vapi, Gujarat, India), and the Zorion BRS (Zorion Medical, Indianapolis, IN, USA). Detailed descriptions of these scaffolds are not yet available.

## Conclusions

BRS is a relatively new technology introduced to address the limitations of the traditional metallic stents. It has been more than 14 years since their first implantation in humans and although there are several BRS available, these devices still have limited applications. This paradox is due to the fact that they have a totally different design and behavior that required extensive study. Evidence from the validation of the second generation BRS indicates that they have overcome the drawbacks of the first generation (eg, rapid bioresorption and device shrinkage) and that they are able to compete with the metallic stents in terms of safety and efficacy. As to whether the dream of a BRS becoming the “workhorse” intracoronary device of the future remains to be answered from the ongoing and upcoming clinical studies.
